# Attitudes and Knowledge Regarding Adipose-Derived Stem Cell Therapy: A Survey of Canadian Orthopedic Surgeons

**DOI:** 10.7759/cureus.86456

**Published:** 2025-06-20

**Authors:** Han Zhi Liu, Vickas Khanna, Olufemi R Ayeni, Naveen Parasu

**Affiliations:** 1 Orthopedic Surgery, McMaster University, Hamilton, CAN; 2 Orthopedics, McMaster University, Hamilton, CAN; 3 Radiology, McMaster University, Hamilton, CAN

**Keywords:** adipose-derived stem cells, autologous stem cell therapy, musculoskeletal disease, orthopedic disease, regenerative medicine therapies

## Abstract

Background

Adipose-derived stem cells (ADSCs) hold therapeutic potential for the treatment of orthopedic conditions. However, no surveys to date have evaluated physicians’ awareness and receptiveness toward these therapies - an essential step in assessing the Canadian healthcare system’s readiness for ADSC implementation, alongside regulatory and technical considerations. We hypothesized a generally low level of exposure to this therapeutic modality, with higher levels of awareness and interest expected among respondents specializing in sports medicine and reconstruction.

Methods

An eight-item questionnaire was distributed to members of the Arthroscopic Association of Canada and the Orthopedic Division at (redacted for blinding) University. Statistical analysis was conducted using mixed-effects logistic regression.

Results

The survey achieved a 12.9% response rate, yielding 50 responses. Most respondents (62%) reported hearing about ADSCs only a few times per year, primarily through scientific journals and colleagues. Only 10% expressed interest in incorporating ADSCs into their future practice. Common barriers included insufficient evidence, high costs, and regulatory limitations. No significant association was found between orthopedic subspecialty and receptiveness to ADSC therapy.

Conclusions

Canadian orthopedic surgeons show limited awareness of, and willingness to adopt, ADSC therapy in clinical practice. Contrary to our hypothesis, no significant differences were observed between subspecialties. Future studies should aim for larger, more representative samples to support more robust conclusions.

## Introduction

Mesenchymal stem cells, a class of adult stem cells, serve as progenitors for various musculoskeletal tissues. They hold therapeutic promise for regenerating cartilage, tendons, and ligaments in orthopedic conditions [1,2]. Traditionally, these cells have been harvested from bone marrow - a method that is invasive, associated with significant pain and infection risk, and often yields insufficient stem cell quantities [3-5]. More recently, adipose-derived stem cells (ADSCs) have emerged as viable candidates for autologous transplantation [3,6,7]. Adipose tissue is widely distributed throughout the body and can be collected via minimally invasive liposuction. Moreover, ADSCs demonstrate favorable regenerative and differentiation capacity in vitro when compared to bone marrow-derived counterparts [7]. Despite these advantages, ADSC use remains limited. In many jurisdictions, such therapies lack regulatory approval, and research into their efficacy for orthopedic applications is still in its early stages [8].

Current studies on ADSCs primarily focus on technical effectiveness. However, the adoption of novel therapies within a healthcare system also depends on factors such as cost, regulation, and provider readiness. There is a notable lack of data on healthcare professionals’ familiarity with, knowledge of, and willingness to integrate ADSC therapies into clinical practice. This study presents the results of a survey assessing Canadian orthopedic surgeons’ knowledge and attitudes toward stem cell therapies for musculoskeletal disorders.

The primary objective was to determine the proportion of Canadian orthopedic surgeons who have prior knowledge or experience with ADSC-related procedures. A secondary objective was to identify potential correlations between surgeons’ subspecialties and their interest in implementing ADSCs in clinical settings. Together, these aims help evaluate the Canadian healthcare system’s readiness to adopt this technology from the perspective of healthcare providers.

We hypothesized that exposure to this therapeutic modality would be generally low among Canadian surgeons, primarily due to limited supporting evidence and regulatory constraints. However, we expected higher awareness and interest among those specializing in sports medicine and reconstruction, as much of the existing ADSC research originates from these subspecialties.

## Materials and methods

Ethics approval was obtained from the Hamilton Integrated Research Ethics Board. A custom survey titled “Adipose-Derived Stem Cells in Orthopedics” was developed, consisting of multiple-choice, short-answer, Likert scale, and checkbox questions. The survey content was curated by a focus group of board-certified physicians actively engaged in ADSC research.

To ensure consistency in understanding terminology, Question 1 included a video demonstration of the autologous ADSC harvesting procedure, as the technique may be referred to by different names in clinical practice (Video 1). The survey was distributed to 387 orthopedic surgeons and residents (Table 1). Contact emails were obtained through the Arthroscopy Association of Canada, the Hamilton Health Sciences hospital network, and the internal communications registry of St. Joseph’s Healthcare. To capture responses from individuals at varying stages of training, all 17 orthopedic residency programs across Canada were contacted. The survey was distributed to the 11 programs that granted permission.

**Video 1:** Mini-liposuction instructional video (three minutes and 20 seconds) This serves as a reference for what the mini-liposuction procedure entails.

**Table 1:** Questionnaire disseminated to respondents

Question	Answer options
1. Watch this mini-liposuction instructional video (see Video 1). Have you performed a mini-liposuction before?	(a) Yes
	(b) No
2. How likely would you be to perform a mini-liposuction procedure?	(a) Very likely
	(b) Likely
	(c) Neither likely nor unlikely
	(d) Unlikely
	(e) Very unlikely
3. Where do you find information about stem cell therapy? (Select all that apply.)	(a) Primary literature
	(b) News/magazines
	(c) Conferences/seminars
	(d) Colleagues
	(e) Patients
	(f) Websites/forums
	(g) Science journals
	(h) Distributors/reps
	(i) Social mixers
4. How often do you hear about the regenerative properties of adipose-derived stem cells?	(a) A few times a day
	(b) A few times a week
	(c) A few times a month
	(d) A few times a year
	(e) Never
5. Would you offer autologous stem cell treatments to your patients?	(a) Definitely would
	(b) Probably would
	(c) Not sure
	(d) Probably would not
	(e) Definitely would not
	(f) Please explain: (open answer question)
6. If not, why? (Select all that apply.)	(a) I don’t believe in it
	(b) Regulatory issues
	(c) Too expensive for patients
	(d) Workplace restriction
	(e) I don’t have time
	(f) Procedure looks too complex
	(g) Other: (open answer question)
7. What are your areas of specialty? (Select all that apply.)	(a) General orthopedics
	(b) Foot and ankle surgery
	(c) Hand surgery
	(d) Hip and knee replacement
	(e) Shoulder and elbow surgery
	(f) Spine surgery
	(g) Orthopedic oncology
	(h) Pediatric orthopedic surgery
	(i) Sports medicine
	(j) Trauma surgery
	(k) Other: (open answer question)
8. How long have you been practicing as an orthopedic surgeon?	(a) I’m currently a trainee, resident, or fellow
	(b) Under 1 year
	(c) 1-5 years
	(d) 5-10 years
	(e) 10+ years

The survey was platformed using Google Forms (Google LLC, Mountain View, California, United States) and designed to be completed within five minutes to minimize respondent attrition. An initial email was sent out to potential respondents, along with follow-up emails at two and six weeks from the initial email, and the survey remained open for six months after initial distribution. To prevent duplicate entries, each email account was limited to one response; the emails were redacted to anonymize data for analysis.

Statistical analysis

Descriptive statistics were calculated using percentage values. To evaluate the association between surgeon subspecialty and the likelihood of implementing ADSCs in future practice, a mixed-effects logistic regression model was employed. This model was appropriate, as each respondent could only select one level of willingness, but could belong to multiple subspecialties.

Willingness to implement ADSCs was treated as a binary variable: “Yes” included Likert responses of “Very likely” or “Somewhat likely,” while “No” encompassed all other responses. The independent variable was surgeon subspecialty, modeled as a fixed effect. Respondents’ subspecialties were split into individual entries, each treated as a random effect using other subspecialties as reference categories in the analysis.

Statistical analysis was conducted in RStudio version 1.2.5033 using the “lme4” package. Data cleaning was performed manually. Incomplete responses were not expected to affect analysis, as all survey fields, except Question 6, were mandatory. Question 6 was intended solely for qualitative insights and was not included in the quantitative analysis.

## Results

A total of 387 participants were contacted, resulting in 50 responses and a response rate of 12.9%. Among the respondents, three (6%) had previously performed a mini-liposuction procedure, while the remaining 47 (94%) had not. When asked about their likelihood of performing mini-liposuctions in the future, three respondents (6%) answered “Very likely,” two (4%) “Likely,” eight (16%) “Neither likely nor unlikely,” 16 (32%) “Unlikely,” and 21 (42%) “Very unlikely.”

Exposure to information about ADSCs came primarily through scientific channels: 32 respondents (64%) cited science journals, 28 (56%) primary literature, and 27 (54%) colleagues. Additional sources included websites and forums (seven respondents, 14%), patients (five respondents, 10%), distributors (three respondents, 6%), and news or magazines (two respondents, 4%). Three respondents (6%) reported no exposure to ADSC information.

Regarding the frequency of exposure to stem cell therapies, two respondents (4%) reported hearing about them “A few times a day,” four (8%) “A few times a week,” eight (16%) “A few times a month,” 31 (62%) “A few times a year,” and five (10%) “Never.”

When asked about their willingness to incorporate ADSCs into their clinical practice if the therapy were approved, two respondents (4%) stated “Definitely would,” six (12%) “Probably would,” 17 (34%) “Not sure,” 18 (36%) “Probably would not,” and seven (14%) “Definitely would not.” The most commonly cited barriers were insufficient evidence (29 respondents, 58%), regulatory concerns (19, 38%), and cost to patients (19, 38%). Other concerns included time commitment (10, 20%), irrelevance to subspecialty (three, 6%), insufficient personal knowledge (three, 6%), and procedural complexity (two, 4%).

Respondents represented a range of orthopedic subspecialties: foot and ankle (six, 12%), adult reconstruction (16, 32%), upper extremity (six, 12%), pediatric (five, 10%), sports medicine (27, 54%), trauma (17, 34%), oncology (three, 6%), and general orthopedic surgery (14, 28%). In terms of career stage, 21 (42%) had been in practice for over 10 years, 13 (26%) for five to 10 years, eight (16%) for one to five years, and eight (16%) were still in training.

**Figure 1:**
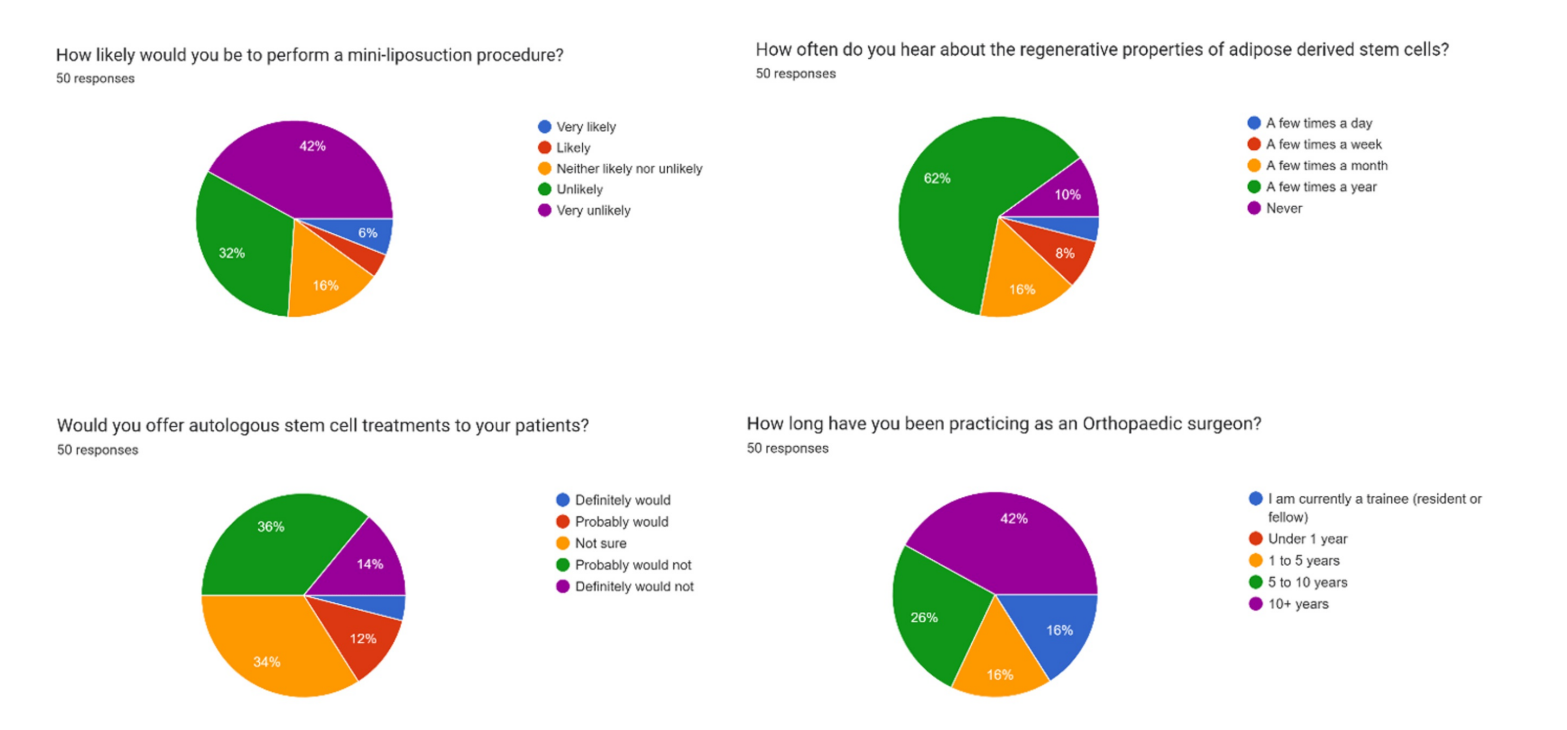
Percentage breakdown of select questionnaire answers

Figure 1 displays a visual breakdown of key response proportions.

A mixed-effects logistic regression analysis revealed no statistically significant associations between orthopedic subspecialty and willingness to implement ADSC therapy in clinical practice (Table 2). Statistical significance was defined as p < 0.05.

**Table 2:** Association between orthopedic subspecialty and willingness to implement ADSCs Each respondent’s subspecialties were treated as separate entries, with subspecialty modeled as a fixed effect on willingness to implement ADSCs in future clinical practice. A mixed-effects logistic regression was used to analyze the data. p-values less than 0.05 were considered statistically significant. ADSC, adipose-derived stem cell

Subspecialty	Estimate	Standard error	z-value	p-value
Sports medicine	-1.38E+01	3.13E+01	-0.441	0.649
Reconstruction	-2.60E+01	1.79E+07	0	1
Trauma	5.23E-01	2.28E+01	0.023	0.982
Upper extremity	-3.85E+01	3.00E+07	0	1
Foot and ankle	4.77E-01	2.51E+01	0.019	0.985
Spine	-1.28E+01	3.48E+05	0	1
Oncology	6.25E-01	2.92E+01	0.021	0.983
Pediatrics	1.09E+00	2.12E+01	0.052	0.959
General orthopedics	3.17E+00	4.27E+01	0.074	0.941

## Discussion

This survey reveals a substantial gap in awareness and readiness among Canadian orthopedic surgeons to adopt ADSC therapy, with only 10% expressing interest in incorporating it into their practices. The hesitancy largely stems from concerns about the sufficiency of current evidence, regulatory hurdles, and cost-related barriers.

A notable insight from the survey is the limited hands-on experience surgeons have with ADSC procurement, as reflected by the majority’s unfamiliarity with performing mini-liposuction procedures. Moreover, the infrequency with which surgeons encounter ADSC-related information, most engaging with stem cell topics only a few times annually, highlights the depth of the knowledge gap. Interestingly, the survey found consistent levels of interest and awareness across orthopedic subspecialties, contradicting the initial hypothesis that certain specialties would show greater familiarity. This uniformity indicates a broad need for enhanced education and stronger evidence across all orthopedic domains, emphasizing the importance of fostering interdisciplinary discussions in regenerative medicine.

Despite promising preclinical and clinical studies demonstrating the safety and efficacy of ADSCs in orthopedic applications [1,9-15], the Canadian orthopedic community remains hesitant to fully embrace these therapies. This reluctance is understandable given the complexities and evolving nature of stem cell research, which requires continued investigation to comprehensively validate its therapeutic potential and safety profile. The demand for further research highlights the need for a robust evidence base that addresses orthopedic surgeons’ concerns, from cellular mechanisms and therapeutic outcomes to long-term efficacy and risk [10,12-14].

Emerging technologies and adherence to Good Manufacturing Practice standards, including innovations such as non-enzymatic autologous ADSC harvesting methods, offer an opportunity to strengthen data reliability and quality in future studies [15,16]. These advances could help overcome existing regulatory and logistical challenges, potentially making ADSC therapies more feasible in clinical settings. By enhancing the safety and efficiency of ADSC processing, such technologies may streamline the transition from research to real-world practice and improve accessibility for patients [16]. Additionally, these advancements may help address one of the primary obstacles in stem cell therapy: regulatory approval.

Given Canada’s healthcare priorities - cost-effectiveness and equitable access - the integration of novel therapies like ADSCs must be carefully evaluated in terms of economic feasibility and policy alignment. Presently, several barriers exist, including the lack of incentive pathways for physician-led innovation, which often demands personal time and financial resources for self-education about ADSCs [17,18]. Another issue is the healthcare system’s focus on cost containment over value creation, making it challenging to justify public investment in emerging technologies [17,19,20]. Provincial policy inconsistencies also hinder unified innovation efforts [17]. Future research must extend beyond clinical outcomes to include health technology assessments and cost-benefit analyses, facilitating informed policymaking and practical strategies for implementation. In this context, Canadian healthcare policy currently prohibits the use of ADSCs for orthopedic treatment. Even in less-regulated sectors like cosmetic medicine, ADSC use remains limited due to exclusion from public health insurance coverage [8]. These factors likely contribute to the low awareness and uncertain adoption prospects observed in this study.

The findings of this survey are limited by the small sample size, which increases the risk of bias and reduces the statistical power of mixed-effects logistic regression. The sample was also limited to respondents from the Arthroscopy Association of Canada and the Orthopedic Division at (redacted for blinding) University, potentially overrepresenting sports medicine specialists and limiting generalizability. Furthermore, the response rate of 12.9% introduces the possibility of response bias, which is a common limitation in online surveys despite follow-up reminders. Due to institutional privacy policies, it was not possible to access all internal registries, and therefore, some individuals may have been counted more than once in the pool of 387 recipients. If so, the actual response rate may be higher than reported. Despite these limitations, this survey is the first of its kind in Canada and serves as a pilot for future research.

## Conclusions

There is limited awareness and exposure to ADSC therapies among Canadian orthopedic surgeons. The combination of insufficient evidence, high costs, and regulatory barriers contributes to the low interest, with only 10% of respondents indicating a willingness to adopt ADSCs in clinical practice. Contrary to initial expectations, no significant differences were found across orthopedic subspecialties in their responses. Addressing these challenges will require further research into ADSC efficacy, greater procedural exposure for surgeons, and accessible educational initiatives. Future studies should aim to improve sample size and diversity to support more robust statistical analyses and to better assess the Canadian healthcare system’s receptiveness to ADSC therapy.
